# Recent Progress in Understanding Plasticity in Neurogenetic Disorders

**DOI:** 10.1155/2012/812472

**Published:** 2012-08-28

**Authors:** Hansen Wang, Cara J. Westmark, Emma Frost, Laurie C. Doering

**Affiliations:** ^1^Faculty of Medicine, University of Toronto, 1 King's College Circle, Toronto, ON, Canada M5S 1A8; ^2^Waisman Center for Developmental Disabilities, University of Wisconsin-Madison, 1500 Highland Avenue, Madison, WI 53705, USA; ^3^Faculty of Pharmacy, University of Manitoba, 750 McDermot Avenue, Winnipeg, MB, Canada R3E 0T5; ^4^Department of Pathology and Molecular Medicine, Faculty of Health Sciences, McMaster University, HSC 1R1, 1280 Main Street West, Hamilton, ON, Canada L8S 4K1

## 1. Introduction

Basic research in the field of neurogenetic disorders will continue to expand our knowledge concerning the development and function of the nervous system. The greatest challenges in the field of neurogenetic disorders are defining the molecular mechanisms by which genetic mutations confer disease risk and phenotype. Neural plasticity involves a series of dynamic cellular events that orchestrate structural and functional alterations in response to experience. The study of neural plasticity provides a basic and powerful approach for unraveling the complexities of the nervous system. Investigations of plastic changes in neurogenetic diseases will shed light on the molecular and cellular mechanisms that govern function of the nervous system, in turn, leading to the discovery of potential therapeutic targets for conditions including mental impairment, neurodegeneration, epilepsy, and autism.

This special issue has targeted the most recent developments in neural plasticity as regards neurogenetic disorders. More than twenty laboratories worldwide have contributed to this special issue. The contributions have showcased the current efforts in understanding neural plastic changes and the underlying molecular and cellular mechanisms in patients and animal models of neurogenetic disorders, including a wide range of neurodevelopmental and neurodegenerative diseases. Additionally, the most promising therapeutic strategies for treating these disorders have been reviewed.

## 2. Plasticity in Neurogenetic Disorders

Fragile X syndrome (FXS), the most common inherited form of mental impairment and autism spectrum disorders (ASDs), is predominantly caused by a CGG repeat expansion in the 5′-UTR of the *FMR1* gene, which encodes the fragile X mental retardation protein (FMRP). With astrocytes playing a pivotal role in the development of synapses in central nervous system, C. Cheng et al. reviewed the current knowledge about the biology of astrocytes and highlighted their involvement in the developmental plasticity of FXS. Martin and Huntsman summarized studies on the mechanisms underlying plasticity deficits in FXS and emphasized that characterizing early developmental deficits in plasticity is fundamental to developing therapies for the disorder. K. Kelley et al. reviewed the studies of repeat-associated miRNAs (ramRNAs) in the transgenic zebrafish model and reported that ramRNA-induced DNA methylation of the *FMR1* 5′-UTR CGG trinucleotide repeat expansion is central to the etiology of FXS. Studies in animal models and patients have indicated that the tetracycline derivative minocycline may hold great therapeutic promise for FXS. Minocycline is thought to act via the inhibition of matrix metalloproteinases (MMPs), the zinc-dependent extracellular proteases involved in tissue remodeling and cell-cell signaling. S. S. Siller and K. Broadie summarized the recent studies on minocycline action in *Drosophila* and mouse FXS models as well as in patients, and discussed a proposed mechanism of minocycline action as an MMP inhibitor.

Rett Syndrome is a progressive neurological disorder caused by mutations in the X-linked *MECP2* gene. MeCP2 was originally known to bind methylated DNA and interact with repressor complexes to inhibit and silence its genomic targets. However, new studies have challenged this idea. R. M. Zachariah and M. Rastegar summarized the current knowledge regarding the molecular function of MeCP2 and pointed out that a collaborative effort between basic scientists and clinicians is required to address the novel and challenging concepts in MeCP2 research and to develop effective therapies for Rett Syndrome. Alterations in dendritic spines have been documented in Rett syndrome; however, C. A. Chapleau et al. reported that the lower dendritic spine density is only apparent in hippocampal CA1 pyramidal neurons of *Mecp2* mutant mice at a presymptomatic stage. This finding suggests that dendritic spine density in hippocampal neurons should not be used as a phenotypic endpoint for the evaluation of therapeutic interventions in symptomatic *Mecp2*-deficient mice and questions the role of MeCP2 in later stages of excitatory synapse and dendritic spine maintenance. The X-linked serine/threonine kinase cyclin-dependent kinase-like 5 (CDKL5) has been associated with early-onset epileptic encephalopathies characterized by intractable epilepsy, severe developmental delay, and the presence of Rett-syndrome-like features. C. Kilstrup-Nielsen et al. reviewed the current state of CDKL5 research with an emphasis on the clinical symptoms associated with mutations in *CDKL5* and the molecular mechanisms of CDKL5 function in neuronal plasticity. 

Down syndrome is a neurodevelopmental disorder caused by triplication of chromosome 21 and is characterized by neurocognitive defects that range from severe intellectual disability to various patterns of neuropsychological deficits. N. Créau reviewed the main molecular and cellular findings observed in mouse models of Down syndrome and described their relationship to disease phenotypes. N. Rueda et al. also summarized studies utilizing Down syndrome mouse models but from the perspective of investigating the neurobiological substrates of mental disability in Down syndrome and testing therapies that could improve cognition. N. Cramer and Z. Galdzicki focused on hippocampal networks which are particularly impacted in Down syndrome, highlighted the neurophysiological changes that reduce the ability of trisomic neurons to undergo neural plastic adaptations, and discussed how altered plasticity may contribute to the cognitive disabilities in Down syndrome patients. Excessive GABAergic neurotransmission dampens hippocampal synaptic plasticity and contributes to cognitive impairments. Treatment with GABAA receptor antagonists results in increased plasticity and improved memory deficits in Down syndrome mice. The selective serotonin reuptake inhibitor fluoxetine can enhance plasticity in the adult rodent brain by attenuating GABAergic inhibition. Unexpectedly, M. Heinen et al. reported that adult-onset fluoxetine treatment does not improve behavioral impairments and even shows adverse seizure and mortality effects in Down syndrome mice raising the possibility of a drug/genotype interaction.

 Angelman syndrome is a neurodevelopmental disorder caused by deletion or loss-of-function mutations in the maternally inherited UBE3A gene and is characterized by severe mental impairment, lack of speech, ataxia, susceptibility to seizures, and unique behavioral features. The UBE3A gene product Ube3a plays an important role in synaptic function and in regulation of activity-dependent synaptic plasticity. N. R. Jana summarized various animal models of Angelman syndrome and discussed how these models provide fundamental insight into understanding the disease biology for potential therapeutic intervention. Tuberous sclerosis complex (TSC) is caused by mutation of either the Tsc1 or Tsc2 genes, which can lead to the disinhibition of mammalian target of rapamycin (mTOR). T. Kirschstein described the animal models that have been established for tuberous sclerosis complex and discussed observed alterations in synaptic plasticity and learning in these models. Charcot-Marie-Tooth disease represents a large group of inherited peripheral neuropathies that involve both motor and sensory nerves and induce muscular atrophy and weakness. P. Juárez and F. Palau summarized the neural and molecular features of Charcot-Marie-Tooth Disease and pointed out that our understanding of the molecular pathways involved in the disease has helped to identify molecular targets for designing novel therapeutic approaches. Copy-number variations (CNVs) in the genome have been identified in several psychiatric disorders including ASDs and schizophrenia. J. Nomura and T. Takumi reviewed the creation of CNV-based animal models of psychiatric disorders and the corresponding neuroanatomical and behavioral abnormalities in these models that provide insight into human neuropsychiatric disorders.

Huntington disease is a neurodegenerative disorder caused by a tandem repeat expansion encoding a polyglutamine tract in the huntingtin protein. R. Vlamings et al. discussed the behavioral, neurophysiological, and histopathological phenotypes of transgenic Huntington disease rats that carry a truncated huntingtin cDNA fragment with 51 CAG repeats under control of the native rat *huntingtin *promoter. Different brain regions, including the hippocampus, cerebral cortex, and striatum, are affected in Huntington disease. M. I. Ransome et al. reviewed the hippocampal abnormalities, in particular the deficits of adult neurogenesis in transgenic Huntington disease mice, and discussed potential mechanisms underlying disrupted hippocampal neurogenesis and how deficits in cellular plasticity may contribute to cognitive and affective symptoms in Huntington disease. 

Spinal muscular atrophy (SMA) is an autosomal recessive neurodegenerative disorder caused by a mutation or deletion in the *survival motor neuron-1* (*SMN1*) gene. L.-K. Tsai summarized the SMN-independent therapeutic targets and strategies with demonstrated potential for the treatment of SMA. Spinal and bulbar muscular atrophy is a polyglutamine disease characterized by progressive muscle weakness and atrophy of the bulbar, facial, and limb muscles pathologically associated with motor neuron loss in the spinal cord and brainstem. Polyglutamine expansion within the androgen receptor is a disease-causing protein that results in spinal and bulbar muscular atrophy. F. Tanaka et al. reviewed current therapeutic strategies for spinal and bulbar muscular atrophy, including those based on the native functions of the androgen receptor. Amyotrophic lateral sclerosis is a neurodegenerative disease principally affecting motor neurons. Besides motor symptoms, patients may develop cognitive disturbances or even frontotemporal dementia, indicating that amyotrophic lateral sclerosis may also involve brain regions outside of the motor regions. F. Trojsi et al. reviewed the current knowledge concerning the neuropsychological and neuropathological sequelae of amyotrophic lateral sclerosis, with a special focus on the neuroimaging findings associated with cognitive change.

Human diseases can now be modeled with relevant cell populations derived from induced pluripotent stem cells (iPSCs) that are generated with techniques that reprogram the somatic cells of patients. H. Wang and L. C. Doering reviewed recent studies using iPSCs to model various neurogenetic disorders and summarized the therapeutic implications of iPSCs, including drug screening and cell therapy for neurogenetic disorders. They highlighted the key issues associated with reprogramming that must be addressed before iPSC technology can translate to the clinic. Brain-derived neurotrophic factor (BDNF) plays essential roles in neuronal development, plasticity, and survival. Investigating the trafficking and release of BDNF is essential for understanding and potentially treating neurological disorders. D. Hartmann et al. summarized multiple techniques to investigate the transport and activity-dependent release of BDNF and their application in neurogenetic disorders. mTOR is a protein kinase involved in many neuronal functions, including dendritogenesis, plasticity, and protein synthesis. The recent literature on the neurological conditions associated with dysregulation of mTOR was covered by T. T. Gipson and M. V. Johnston. In addition, clinical trials for neurogenetic disorders with abnormalities in synaptic plasticity, and mTOR signaling were discussed.

## 3. Conclusion

Over the past decade, we have witnessed a dynamic expansion in the study of neurogenetic disorders. This special issue is by no means exhaustive of the research in this exciting field. We hope that the papers published herein will give our readers a broad sense of the recent progress in the field of plasticity in neurogenetic disorders as well as inspire future work that will provide a better understanding of the disease mechanisms and eventually lead to more effective treatments. 

